# Exploring how and why Care Groups work to improve infant feeding practices in low- and middle-income countries: a realist review protocol

**DOI:** 10.1186/s13643-020-01497-1

**Published:** 2020-10-10

**Authors:** Pieternella Pieterse, Anne Matthews, Aisling Walsh, Ellen Chirwa

**Affiliations:** 1 School of Nursing, Psychotherapy and Community Health, Dublin City University, Dublin, Ireland; 2 Department of Public Health and Epidemiology, Royal College of Surgeons in Ireland, Dublin, Ireland; 3 Kamuzu College of Nursing, University of Malawi, Blantyre, Malawi

**Keywords:** Realist review, Care Groups, Exclusive breastfeeding, Infant nutrition, Malawi

## Abstract

**Background:**

Within our inquiry into the implementation of breastfeeding policy in Malawi, Care Groups have been mentioned as a means to improve maternal and child health and nutrition outcomes. The ‘Care Group model’ is an approach primarily used in international development settings, whereby social and behaviour changes are promoted through supported peer-to-peer (mostly mother-to-mother) knowledge sharing. The aim of most Care Groups is to promote improved infant nutrition, improve hygiene and increase the number of children who are fully vaccinated and exclusively breastfed for the first 6 months. The behavioural changes promoted by Care Groups (such as safe infant feeding, frequent hand washing, consistent mosquito net usage, providing suitable complementary foods from 6 months old) have the potential of averting preventable deaths particularly among children under five.

While a variety of approaches are used to promote improved health and nutrition for children under five, the Care Groups model was best known and frequently referenced during our discussions with key stakeholders regarding the delivery at community level of Malawi’s National Multi-Sector Nutrition Policy 2018-2022. A better understanding of how Care Groups achieve their social and behaviour change results and how community-based efforts are sustained can potentially help to ensure more effective planning and budgeting for Care Group interventions and enable greater sustainability and increased coverage of infant feeding support countrywide. This realist review is designed to improve our understanding of how, why, to what extent and under what circumstances Care Groups improve infant feeding practices in low- and middle-income countries (LMICs).

**Methods and analysis:**

A realist review is a theory-driven approach to evidence synthesis. To undertake this realist review, we will gather evidence by conducting peer-reviewed and grey literature database searches in order to find peer reviewed articles, programme guidelines and evaluation reports, among other texts, associated with the implementation of Care Groups in low- and middle-income countries. Our review process has five key steps: (1) locating existing theories; (2) searching for evidence in literature; (3) selecting articles and other suitable evidence; (4) extracting data, identifying configurations of context-mechanism-outcomes; and (5) synthesising the evidence, drawing conclusions.

**Discussion:**

The results of this realist review will be written up according to RAMESES guidelines and disseminated through a stakeholder workshop in Malawi, through conference presentations and peer-reviewed publications. It is intended to improve the understanding of the potential and limits of working through Care Groups globally and among relevant Malawi Ministry of Health staff and the donor and NGO community, both internationally and within Malawi. This systematic review protocol has been submitted for registration on the PROSPERO database (receipt number: 170261).

## Background

### What are Care Groups and why are they considered to improve infant feeding practices and related child health outcomes in low- and middle-income countries?

Many children under five die of preventable and easily treatable causes every year. In low- and middle-income countries (LMICs), as many as 65 children per 1000 live births are thought to have died before their fifth birthday in the poorest households in 2016 [1]. The Sustainable Development Goals (SDGs) call for an end to preventable deaths due to treatable illness of newborns and children under five by 2030, with all countries aiming to reduce under-5 mortality rates to no more than 25 deaths per 1000 live births [2]. Efforts to achieve this focus not only providing better and more accessible healthcare, but also on preventing children from getting sick through a range of public health ‘social and behaviour change’ interventions [3, 4]. One such approach to promoting social and behavioural change is through the use of Care Groups. The approach was developed in Mozambique in 1995 and has since been implemented by non-governmental organisations (NGOs) in almost 30 countries, supported by government and international aid donors (https://caregroupinfo.org/about-us/).

The Care Group approach is based on the principle of peer-to-peer (usually mother-to-mother) health promotion, targeted at women of reproductive age, and their families, to transfer key health messages regarding their own health and that of their newborn babies and young children. Care Groups are composed of a lead volunteer, usually a mother, who spreads basic health information to a maximum of 12–16 women/families in her community [5]. The lead mothers (Care Group Volunteers) are provided with training on a single issue every 2–4 weeks, after which they teach key messages regarding this particular issue to their respective group members. The lead mothers are trained by a promotor, who is responsible for up to nine Care Groups, reaching approximately 150 persons per promotor. According to the Care Group Manual, published by the Food Security and Nutrition Network Social and Behavioral Change Task Force in 2014, 100% of households in an intended group or community should be targeted and a Care Group initiative should attain at least 80% monthly attendance [3], p 34:

‘In order to create a ‘new social norm’ (not one person changing behavior, but many encouraging each other) a program needs to reach most or all of the households with women who could get pregnant, are currently pregnant or have young children (under 5)’

Evidence suggests that Care Groups can often be an effective approach to changing families’ behaviours, which may reduce the frequency and severity of childhood illnesses [5, 6]. However, little clarity exists regarding how and why Care Groups work and what the reasons are for this approach working well in some places, under certain circumstances, promoting certain types of behaviours, and being much less effective and/or sustainable in other settings.

### Identifying the need for research on Care Groups

This research started as an inquiry into the implementation of breastfeeding policy in Malawi, with a specific focus on promoting and supporting exclusive breastfeeding for the first 6 months. During an initial scoping visit, with the objective of narrowing the focus of our research on the community-level implementation of Malawi’s breastfeeding policy, it became clear that the Care Group approach warranted further investigation. The Ministry of Health and international aid donors mentioned the existence of Care Groups on several occasions. It was clear that among many stakeholders, the assumption was that Care Groups ensure that community-level breastfeeding support is provided to every woman who needs it. Certain members of the international aid donor community acknowledged that ‘more research needs to be done’, into both the coverage that currently existing Care Groups in Malawi provide, in terms of breastfeeding and additional infant nutrition support, and into their sustainability.

The most recently available DHS survey data for Malawi (2015–2016) show that 61% of children between the ages of 0 and 6 months were exclusively breastfed [7]. However, other studies [7–9] and regional trends suggest that while exclusive breastfeeding remains common for newborns, it is much less prevalent for infants who are older than 3 months. At this stage, continued exclusive breastfeeding can provide babies with enormous benefits in terms of improved nutrition and protection from diarrhoeal diseases [10, 11]. Continued support to encourage exclusive breastfeeding for the full 6 months therefore remains a policy priority for Malawi’s Ministry of Health.

Our overall research aims are to produce policy guidance on effective exclusive breastfeeding support. One aspect of this relates to questions regarding how Care Groups achieve their outcomes and if they are effective in improving exclusive breastfeeding rates. In addition, to understand how scaling up of Care Groups could be achieved and made sustainable, it is essential to gain a greater understanding of how Care Groups work, what the mechanisms are by which Care Group interventions are seen to result in positive, and sustained outcomes. To meet this objective, we will carry out a realist review of Care Groups in LMICs. Unlike other approaches to the synthesis of evidence, realist reviews allow the researchers to focus questions such as how, why, for whom and to what extent a certain intervention may contribute to generating particular outcomes, which is exactly the objective of this research.

### What are Care Groups?

A scoping search, based on the realist review approach, supported the formulation of our research strategy, this protocol, and provided us with a basic understanding about Care Groups: The Care Group approach was developed in 1995 by staff from the charitable organisation World Relief.^1^ World Relief and a second American NGO, Food for the Hungry, have been at the forefront of Care Group implementation, often funded by the US Agency for International Development. In the past decade, Care Groups have been used in at least 27 countries (https://caregroupinfo.org/about-us/). The Care Group approach might now be considered among a range of community-based health support approaches that is relatively common within international development practice. The use of community-based healthcare interventions, supported by international development actors, has grown dramatically since the 2006 World Health Organisation report ‘Working together for health’ raised the alarm in relation to a critical shortage of health service providers and cautioned that in the future, the use of health volunteers and community health workers would become a reality [11].

The Care Group approach contains many of the properties of complex interventions: ‘Complex interventions depend on human behaviour and their active ingredients tend to enable people to do the right thing at the right time or constrain them from doing something’ [12]. However, exactly how the Care Group approach of ‘social and behaviour change promotion’ achieves its results is not entirely obvious. There does not seem to be an overarching theory of change, which could have pointed to how the various components are expected to lead to the desired change and why. The 2014 Care Group manual [3] seems to suggest that individual components of the approach are based on existing substantive theories such as social norms theory, protection motivation theory, expectancy theory and others. Nevertheless, these theories combined still lack the explanatory power of why Care Groups work, for certain people, under certain circumstances.

### Realist reviews

A realist review is a theory-driven, interpretive approach to the synthesis of evidence. It seeks to interrogate the theories that underpin the intervention that is being studied, which is Care Group interventions, in this case. Realist reviews aim to produce explanatory analyses, that is, what works, for whom, in what circumstances, in what respects [13]. The ‘outcome’ of a realist review is one, or a series of, refined programme theory/ies; a more refined understanding of which components of a certain intervention may trigger a certain response, under certain conditions. Realist research focuses on identifying how, why, when, for whom and to what extent interventions such as Care Groups change or manipulate contexts (C), which then trigger the mechanisms (M) which can lead to outcomes (O). A realist review is in effect a combing of all relevant literature in order to establish what exactly happens when a certain complex intervention is implemented. Given that it seems clear that Care Groups work, under certain conditions, but that it remains unclear exactly how, for whom, why, to what extent and under which circumstances, using a realist review approach is ideally suited for this research.

Distilling the various possible CMO configurations from the literature will allow us to test our initial programme theories and refine or reject these, based on the patterns, so-called demi-regularities [14] that may be encountered during our data synthesis and analysis phase.

### Reference group

It is common in realist reviews to work with a stakeholder reference group whose members can advise throughout the review process. The stakeholders who will comprise the reference group for this review are a mix of individuals who have an interest in using the findings of this research. They will include individuals working for the Ministry of Health in Malawi, those working for international aid donors in Malawi, those who are experts regarding their practical implementation of Care Group interventions both in Malawi and globally and those who are nutrition and/or public health experts in Malawi. They will be consulted on a number of occasions throughout this research, either in person, via email or teleconference, Skype, etc. We will establish this group as soon as the review commences.

A realist review can produce important information about how various components of Care Group interventions work, why they do so, for whom they work best/not and under which circumstances, thereby enabling stakeholders and practitioners to make informed decisions about the best structure and processes for future implementation.

**Fig. 1:**
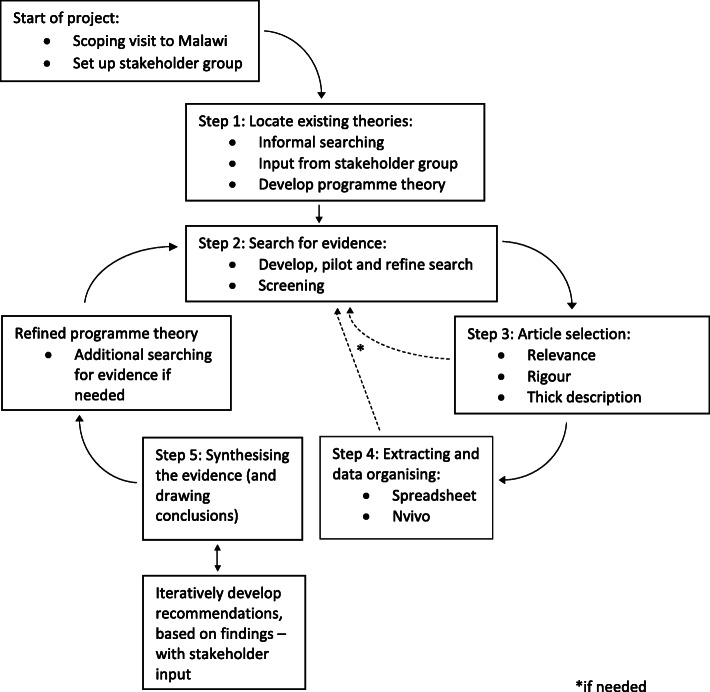
Flow diagram of realist review process

For our realist review, we will follow the steps for realist and meta-narrative evidence synthesis, as outlined by Greenhalgh et al. [15] and visualised in Fig. 1, based on an original flow diagram by Wong et al. [16].

## Methods

### Aim

The aim is to explore how, why, when, in what contexts and to what extent Care Group interventions achieve their impact and what mechanisms ensure their sustainability.

### Objectives

To produce policy guidance on effective exclusive breastfeeding support, we will need to be able to explain how and why Care Groups work. In order to do so, we will conduct this realist review taking the following steps: (1) developing initial programme theories, based on how Care Groups appear to work; (2) carry out an analysis of the selected peer reviewed and grey literature, and, where relevant, establish new theories; and (3) test these, based on the evidence we find within the texts. (4) During this process, we will seek out the context-mechanism-outcome configurations that can be deduced from the literature (5) and use the most often repeated CMO configurations, so-called demi-regularities, to produce explanatory theories of causation which will take the form of CMO configurations which are middle range in nature.

Factors contributing to Care Groups achieving their outcomes will be documented, along with the contextual factors that may influence their sustainability. From the initial scoping search, examples include factors such as suitable communication materials, Care Group volunteers’ workload and any rewards that may be provided to Care Group volunteers as encouragement—such details and their possible impact will all be documented. The outcome of this study will be the identification of the optimal conditions for the successful establishment and sustainability of Care Groups in Malawi. As this review is based on literature from a range of LMICs, we expect it to produce findings that are transferable beyond Malawi, to other settings where similar contexts exist.

### Review questions

1. What are the mechanisms and contexts by which Care Groups achieve social and behavioural change?
2. What are the mechanisms and contexts which determine whether Care Groups are sustainable beyond their initial establishment and life span of an NGO's programme support?^2^
3. What are the mechanisms and contexts by which Care Group interventions achieve the outcome of increasing exclusive breastfeeding practices in LMICs?

### Study design

#### Step 1: Develop initial programme theories

The realist review began with a scoping search that has allowed the research team to become familiar with the literature, and to develop initial programme theories, based on a reading of the academic papers and grey literature found during the initial search (e.g. [3, 5, 6, 17]). A librarian is being consulted to assist with literature searches throughout the research.

As a group of researchers, we explored the question regarding ‘which programme theories could be ascribed to Care Groups?’. Care Groups are predominantly used to promote optimal infant health and nutrition. Care Groups seem to work because they rely on ordinary people, mainly women with young children, to promote health messages and behaviours (such as hand washing, exclusive breastfeeding) among other women in her community, who accept them because they trust the messenger. Care Group promotors meet with leader mothers every 2–4 weeks to pass on a new message and visual aids to make sure the information is clear. The Care Group leader mothers seem happy to carry out these tasks with little or no incentives and devote a significant amount of time to her Care Group tasks [5, 6]. Other community members appear to welcome the leader mothers and accept their advice, many even change their behaviour. Some pre-implementation research is usually conducted at the start of a programme, allowing the implementing agency to gather sufficient local community knowledge to ensure that all of its behavioural change messages are culturally and practically acceptable. Results from Care Group interventions have included reductions in infant mortality rates and improved nutritional status among infants in target communities [18].

#### Step 2: Undertake literature searches

We completed a search term template (Additional file 1: Appendix 1) covering each concept/component of our topic. After conducting a scoping literature search (step 1), we developed a comprehensive search strategy. A sample search string for CINAHL is included as Additional file 1: Appendix 2. We will undertake searches iteratively, with the following components:

1. Electronic database searching, using keywords Care Group, Maternal and Child Health, MCH, nutrition and countries/continents of relevance (see Additional file 1 for search terms and sample initial search strategy), targeting CINAHL, EMBASE, MEDLINE, the Cochrane Library, Web of Science, ASSIA and any other relevant databases. A search log will be maintained for transparency.
2. Hand searching citations contained in the reference lists of included papers;
3. Grey literature searching, using grey literature specialist data bases such as OpenGrey, but also websites that are sector specific, relating to Care Groups, such as https://caregroupinfo.org/ and nutrition, such as the Emergency Nutrition Network (ENN) data base. We have drafted specific search parameters for the grey literature search, included in Additional file 1: Appendix 3.
4. Contacting authors; contacting site managers of specialist websites such as caregroupinfo.org

The search for evidence in a realist review is iterative and may be refocused (based on the identified sources) as the review evolves.

#### Step 3: Sorting data based on inclusion criteria and procedures

Two researchers will screen all papers by title and abstract (where possible) in the first instance, to ensure that the correct inclusion criteria were applied. The papers that remain after the initial selection will be read in full by two researchers, who will select suitable papers based on relevance, using the question ‘can this document provide data that informs programme theory development and refinement?’. Any disagreements will be settled through discussion with the extended research team.

Realist reviews incorporate a range of literatures, qualitative, quantitative, peer-reviewed and grey literature. Our scoping search of the literature suggests that in relation to Care Groups, the latter is likely to be available in larger quantities than peer-reviewed texts. Different approaches to quality assessments of the literature may therefore be needed during this review. We will judge the data on its trustworthiness (Wong [17]) and use Hardwick et al.’s approach [19] to assess data quality, based on how well it meets the explanatory needs of the review. Each piece of evidence will be judged on how relevant it is to theory building, theory refining and theory testing. Based on the range of texts found during our scoping search, an additional set of inclusion and exclusion criteria has been formulated:

Inclusion:

- Articles/reports etc. where there is some focus on and findings related to the implementation of a Care Group approach
- Articles/reports etc. where concrete methods and/or the implementation processes, as practiced in the field, have been described—with references to when and where (see above for comment on rigour/quality)
- Articles/reports etc. where findings are presented from Care Group fieldwork

Exclusion:

- Text that provides only general guidance on how to set up/implement Care Groups
- Text that makes general statements about what Care Groups have achieved without these claims being linked to a particular intervention (not enough detail, no references etc.)

Search results will be presented in a PRISMA flow diagram.

#### Step 4: Extracting and organising data

The full texts of all included papers will be uploaded to NVivo12 (qualitative data analysis software).

Relevant sections of texts relating to Care Group contexts, mechanisms and/or their relationships to outcomes will be coded in NVivo12. This coding will be both deductive, using codes created in advance of data extraction and analysis, based on our initial programme theory; and inductive, with codes being created to categorise data found in the included studies. Each new data point will be used to refine the theory, if appropriate, and as the theory is refined, the included studies will be revisited to search for further data that may be relevant to the revised theory, but which may have been missed initially. Refinement of the theory will lead to a more nuanced understanding of the subject; in the case of Care Groups, it may lead us a greater understanding of the size of the impact of frequency of training, the quality of flip chart teaching aids, the involvement of community leaders, husbands, etc. The characteristics of all included documents will also be entered separately into an Excel spreadsheet, using an adapted Arksey and O’Malley framework [20] to be able to create an overview of the various types of literature that have been used for this review.

#### Step 5: Data analysis and synthesis

Our data analysis and subsequent synthesis aims to provide clarity regarding the contexts (C) in which Care Groups operate, the mechanisms (M) that are activated when these contexts are combined with the Care Group intervention, and we seek to find clarity regarding which outcomes (O) are produced by each distinct C-M combination. Using all four types of reasoning commonly employed in realist research, induction, deduction, retroduction and adjudication will allow us test our initial programme theory and the various possible C-M-O configurations from the literature, and refine or reject these, based on the demi-regularities that may be encountered during our data synthesis and analysis phase. This will allow us to build our final programme theory.

## Discussion

### Importance of the research

The findings of this research will be shared with our stakeholders, as well as with a wider group of individuals who will be invited to attend a feedback workshop in Malawi. This realist review will furthermore be the basis for the design of a Realist Evaluation of ongoing Care Group interventions in Malawi, which should jointly provide ‘the kind of rich, detailed and highly practical understanding of complex social interventions which is likely to be of much more use to them when planning and implementing [Care Groups] programmes’ [13], specifically with a focus on exclusive breastfeeding support. The realist review will be also published in a peer-reviewed journal, and it is envisaged that this will contribute to filling the knowledge gap that exist regarding the benefits and draw-backs of Care Group approaches globally. It will also contribute to the growing number of realist reviews that focus on interventions that are unique to health and nutrition in low- and middle-income countries.

## Supplementary information

**Additional file 1: Appendix 1.** Search terms. **Appendix 2.** Initial CINAHL (all fields) search. **Appendix 3.** Grey literature search parameters.

## Data Availability

Data sharing is not applicable to this article as no datasets were generated or analysed during the current study.

## Abbreviations

CG: Care Group
LMICs: Low- and middle-income countries
NGO: Non-governmental organisation

## Footnotes

1 https://caregroupinfo.org/

2 This can be a mix of training, supportive supervision and some small gifts or certificates to motivate volunteers.
