# Supplementary material for: Exploring how and why Care Groups work to improve infant feeding practices in low- and middle-income countries: a realist review protocol
Source: Syst Rev. 2020 Oct 10;9:237. doi: 10.1186/s13643-020-01497-1 (PMC7548036; doi:10.1186/s13643-020-01497-1)
Supplement: Supplementary file 1 — Additional file 1: Appendix 1. Search terms. Appendix 2. Initial CINAHL (all fields) search. Appendix 3. Grey literature search parameters. [file 13643_2020_1497_MOESM1_ESM.docx]

**Additional file 1:**

**Appendix 1: Search terms**

**Search parameters:**

Texts published after 1995

Text in English

| **Concept 1** | **Concept 2** | **Concept 3** |
| --- | --- | --- |
| Care Group | Infant feeding practices | Low and Middle Income Countries (LMICs) |
| Care Group  OR  Care Groups | Maternal newborn and child health  OR  Nutrition  OR  Health  OR  Breastfeeding  OR  Infant feeding  OR  Maternal  OR  Child  OR  Infant  OR  Newborn  OR  MCH  OR  MNCH  OR  Survival | Low and Middle Income Countries  OR  LMIC*  OR  Developing countries  OR  Africa  OR  Asia  OR  South America  OR  Central America  OR  List of individual LMI countries- See <https://data.worldbank.org/income-level/low-and-middle-income>] |

**Appendix 2: Initial CINAHL (all fields) search:**

1. “CARE GROUP*”

AND

2. Health OR survival OR Maternal OR Child OR Infant OR Newborn OR Nutrition OR "Infant feeding" OR Breastfeeding OR Feeding OR Maternal newborn and child health OR MNCH OR MCH OR Maternal-Child Health

AND

3. Low and Middle Income Countr* OR LMIC* OR "Developing countries" OR Africa OR Asia OR "South America" OR "Central America" OR AFGHANISTAN OR ALBANIA OR ALGERIA OR AMERICAN SAMOA OR ANGOLA OR ARGENTINA OR ARMENIA OR AZERBAIJAN OR BANGLADESH OR BELARUS OR BELIZE OR BENIN OR BHUTAN OR BOLIVIA OR BOSNIA HERZEGOVINA OR BOTSWANA OR BRAZIL OR BULGARIA OR BURKINA FASO OR BURUNDI OR CABO VERDE OR CAMBODIA OR CAMEROON OR CENTRAL AFRICAN REPUBLIC OR CHAD OR CHINA OR COLOMBIA OR COMOROS OR CONGO OR COSTA RICA OR COTE D’IVOIRE OR CUBA OR DJIBOUTI OR DOMINICA OR DOMINICAN REPUBLIC OR ECUADOR OR EGYPT OR EL SALVADOR OR EQUATORIAL GUINEA OR ERITREA OR ESWATINI OR ETHIOPIA OR FIJI OR GABON OR GAMBIA OR GEORGIA OR GHANA OR GRENADA OR GUATEMALA OR GUINEA OR GUINEA-BISSAU OR GUYANA OR HAITI OR HONDURAS OR INDIA OR INDONESIA OR IRAN OR IRAQ OR JAMAICA OR JORDAN OR KAZAKHSTAN OR KENYA OR KIRIBATI OR KOREA OR KOSOVO OR KYRGYZ REPUBLIC OR LAO OR LEBANON OR LESOTHO OR LIBERIA OR LIBYA OR MADAGASCAR OR MALAWI OR MALAYSIA OR MALDIVES OR MALI OR MARSHALL ISLANDS OR MAURITANIA OR MAURITIUS OR MEXICO OR MICRONESIA OR MOLDOVA OR MONGOLIA OR MONTENEGRO OR MOROCCO OR MOZAMBIQUE OR MYANMAR OR NAMIBIA OR NAURU OR NEPAL OR NICARAGUA OR NIGER OR NIGERIA OR NORTH MACEDONIA OR PAKISTAN OR PAPUA NEW GUINEA OR PARAGUAY OR PERU OR PHILIPPINES OR ROMANIA OR RUSSIAN FEDERATION OR RWANDA SAMOA OR SAO TOME AND PRINCIPE OR SENEGAL OR SERBIA OR SIERRA LEONE OR SOLOMON ISLANDS OR SOMALIA OR SOUTH AFRICA OR SOUTH SUDAN OR SRI LANKA OR ST LUCIA OR ST VINCENT AND THE GRENADINES OR SUDAN OR SURINAME OR SYRIAN ARAB REPUBLIC OR TAJIKISTAN OR TANZANIA OR THAILAND OR TIMOR-LESTE OR TOGO OR TONGA OR TUNISIA OR TURKEY OR TURKMENISTAN OR TUVALU OR UGANDA OR UKRAINE OR UZBEKISTAN OR VANUATU OR VENEZUELA OR VIETNAM OR WEST BANK AND GAZA OR YEMEN OR ZAMBIA OR ZIMBABWE

4. 1 AND 2 AND 3

**Appendix 3: Grey literature search parameters**

Searching for grey literature that is relevant to this review will be carried out in the following manner:

- Google and Google scholar will be avoided, due to the fact that the words ‘care’ and ‘group’ are extremely common. Even both words together produce too many references to check.
- Instead we will use platforms such as OpenGrey and specialist websites that focus on either Care Groups ([www.caregroupinfo.net](http://www.caregroupinfo.net)) or Nutrition ([www.ennonline.net](http://www.ennonline.net)) to find grey literature or find references to particular Care Group interventions that can then be searched for using Google.

To keep the total number of documents included for review to a manageable level, the researchers will start by including grey literature from a limited number of years, starting with 2015-2020, to which our inclusion and exclusion criteria will be applied. After the full texts of the included papers have been read, the range may be expanded and additional grey literature included, if the researchers believe that ‘saturation’ has not yet been reached^[[1]](#footnote-1)^, and many of the initial programme theories have been neither confirmed not refuted by the included papers.

1. Geoff Wong, Gill Westhrop, Ray Pawson and Trish Greenhalgh (2013) Realist Synthesis, RAMESES Training Material, p. 30. [↑](#footnote-ref-1)
